# Treatment Changes and Prognoses in Patients with Incident Drug-Induced Parkinsonism Using a Korean Nationwide Healthcare Claims Database

**DOI:** 10.3390/jcm12082860

**Published:** 2023-04-13

**Authors:** Siin Kim, Hae Sun Suh

**Affiliations:** 1 College of Pharmacy, Kyung Hee University, Seoul 02447, Republic of Korea; siin@khu.ac.kr; 2 Institute of Regulatory Innovation through Science, Kyung Hee University, Seoul 02447, Republic of Korea; 3 Department of Regulatory Science, Graduate School, Kyung Hee University, Seoul 02447, Republic of Korea

**Keywords:** antipsychotics, drug-induced parkinsonism, metoclopramide, parkinsonism, prognosis, treatment pattern

## Abstract

This retrospective cohort study assessed treatment changes and prognoses after incident drug-induced parkinsonism (DIP). We used the National Health Insurance Service’s National Sample Cohort database in South Korea. We selected patients diagnosed with incident DIP and given prescriptions to take offending drugs (antipsychotics, gastrointestinal (GI) motility drugs, or flunarizine) for a period of time that overlapped with the time of DIP diagnosis during 2004–2013. The proportion of patients experiencing each type of treatment change and prognosis was assessed for 2 years after DIP diagnosis. We identified 272 patients with incident DIP (51.9% of patients were aged ≥ 60 years and 62.5% of them were women). Switching (38.4%) and reinitiation (28.8%) were the most common modifications in GI motility drug users, whereas dose adjustment (39.8%) and switching (23.0%) were common in antipsychotic users. The proportion of persistent users was higher among antipsychotic users (7.1%) than that among GI motility drug users (2.1%). Regarding prognosis, 26.9% of patients experienced DIP recurrence or persistence, the rate being the highest in persistent users and the lowest in patients who discontinued the drug. Among patients with incident DIP diagnoses, the patterns of treatment change and prognosis differed across the types of offending drugs. Over 25% of patients experienced DIP recurrence or persistence, highlighting the need for an effective strategy to prevent DIP.

## 1. Introduction

Drug-induced parkinsonism (DIP) is a parkinsonian syndrome induced by medications that inhibit dopamine function in the brain categorized as secondary parkinsonism [1,2]. Compared with Parkinson’s disease, DIP is characterized by acute, symmetrical, and reversible symptoms [3]. However, it is difficult to completely distinguish the two diseases in clinical practice, leading to misdiagnosis and inappropriate management [4].

Antipsychotics, gastrointestinal (GI) motility drugs, dopamine depleters, and calcium channel blockers are well-known offending drugs which pose a high risk of developing DIP [1,2,3,5]. Although typical antipsychotics have been known to pose a high risk of developing DIP, atypical antipsychotics and GI motility drugs, especially levosulpiride and metoclopramide, also have a risk level comparable to that of typical antipsychotics [6].

As DIP is generally under-recognized in clinical settings, its true incidence may be much higher [7,8]. Nevertheless, DIP is the second most common cause of parkinsonism after Parkinson’s disease [9]. In South Korea, the incidence of DIP increased from 2012 to 2015, reaching 13.9 per 100,000 person-years, with a particularly high incidence among middle-aged individuals [10]. A retrospective study using nationwide hospital-based data in China reported a higher comorbidity burden and hospitalization expenses among patients with secondary parkinsonism than that among patients with Parkinson’s disease, suggesting the considerable economic burden of secondary parkinsonism [11]. As one of the main adverse drug reactions related to antipsychotics, DIP can result in decreased health-related quality of life and working memory performance in patients with schizophrenia [12,13]. In these patients, DIP is associated with increased healthcare resource utilization (e.g., hospitalization and emergency room visits) and higher costs of care, and can hinder the optimal treatment with antipsychotics [14,15]. Moreover, patients with DIP had a significantly increased risk of developing Parkinson’s disease, which can add the burden of managing of Parkinson’s disease for patients with DIP [16].

Avoiding offending drugs is the most effective way of preventing or treating DIP [1,8]. DIP is generally resolved within 4 months if patients stop taking the offending drugs [17]. However, some patients may be unable to avoid taking offending drugs, as the expected clinical benefits may outweigh the risk of DIP [8]. Furthermore, some patients may experience persistent symptoms even after the discontinuation of the offending drugs [8,17]. Considering the growing number of patients being prescribed offending drugs, the effective management of DIP is essential to minimize the societal and economic burden of adverse drug reactions [18]. To seek an effective strategy to manage DIP, it is important to understand the current treatment patterns and prognoses in patients with DIP. However, the evidence for the management status of DIP is very scant, and the evidence reported so far is based on old data or a limited patient group [17,19].

Therefore, this study aimed to explore the status of treatment changes and prognoses after the occurrence of incident DIP using a representative Korean national claims database. We followed-up on patients who had experienced incident DIP for 2 years, and assessed the treatment changes and prognoses associated with three major types of offending drugs: antipsychotics, GI motility drugs, and flunarizine.

## 2. Materials and Methods

### 2.1. Study Design and Data Source

We conducted a retrospective cohort study using the National Health Insurance Service’s National Sample Cohort (NHIS-NSC; NHIS-2018-2-238) database of South Korea from 1 January 2002 to 31 December 2015. The NHIS provides single-payer coverage to the entirety of Korea’s population through the National Health Insurance program (97%) and Medical Aid program (3%) [20]. The National Health Insurance program covers employees and self-employed individuals, while the Medical Aid program is administered by the Korean government as a public assistance scheme that safeguards the basic livelihood of individuals with low incomes by offering healthcare services. The NHIS-NSC database comprises longitudinal information on demographic characteristics, diagnoses, healthcare service uses, and healthcare costs among a representative sample (approximately 1 million citizens) from the general Korean population [21]. This study was exempt from a review from the Institutional Review Board (IRB) of Pusan National University (PNU IRB/2016_29_HR).

### 2.2. Selection of Incident DIP Cases

**Figure 1:**
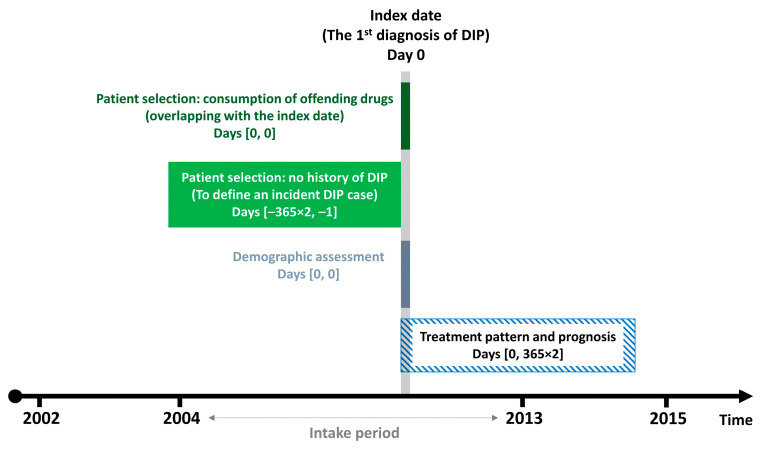
Study design scheme. Abbreviations: DIP, drug-induced parkinsonism.

We identified individuals diagnosed with DIP (International Classification of Diseases, Tenth Revision (ICD-10) codes G21.1 (other drug-induced secondary parkinsonism) or G25.1 (drug-induced tremor)) as the primary or secondary diagnosis between 1 January 2004 and 31 December 2013 (Figure 1). The earliest date of DIP diagnosis was defined as the index date. At least one outpatient prescription for the offending drug was required to be for a time period that overlapped the index date. Offending drugs included typical and atypical antipsychotics (amisulpride, aripiprazole, chlorpromazine, clozapine, haloperidol, olanzapine, paliperidone, perphenazine, pimozide, quetiapine, risperidone, sulpiride, and ziprasidone), GI motility drugs (clebopride, domperidone, itopride, levosulpiride, metoclopramide, and mosapride), and flunarizine, which were available in South Korea during the study period. To identify incident DIP cases, patients with DIP diagnoses preceding the index date by up to two years were excluded from the study.

### 2.3. Outcome Measures

To explore the treatment changes in the consumption of offending drugs and prognoses of DIP, individuals with incident DIP were followed-up for 2 years from the index date. The first treatment change for each patient was defined based on the pattern in which the offending drugs were prescribed (Table 1). The types of treatment changes included discontinuation, dose adjustment, persistent use, reinitiation, and switching to other offending drugs. Each treatment change was mutually exclusive. A continued use of offending drugs was defined as having consecutive prescriptions with a gap period of ≤60 days. Replacing drugs with drugs from the same therapeutic class (i.e., antipsychotics, GI motility drugs, and flunarizine) was considered switching, while replacing drugs with drugs from other therapeutic classes was considered temporary discontinuation. For example, replacing metoclopramide with domperidone was considered an act of switching to other offending drugs, while replacing metoclopramide with nizatidine (histamine type 2 receptor antagonists) was considered an act of temporary discontinuation. As a switch to other offending drugs was required to occur within consecutive prescriptions, a switch after a temporary discontinuation was regarded as reinitiation in this study.

The first prognosis for each patient was defined based on the pattern of DIP diagnosis, and categorized into persisting DIP, DIP recurrence, and DIP remittance. An episode of DIP was considered a remittance if no further DIP was diagnosed over 4 months based on the published literature [8,17]. In other words, consecutive prescriptions with a DIP diagnosis within 4 months were considered part of the same episode of DIP.

We estimated the proportion of patients with each type of treatment change and prognosis. The proportion was calculated among all patients with incident DIP and for each therapeutic class (i.e., antipsychotics, GI motility drugs, and flunarizine). We estimated the time from the index date to the first treatment change, DIP remittance, and DIP recurrence. The time to event was analyzed in patients who experienced each type of event. For example, we estimated the time to remittance in patients who had previously experienced remittance during the follow-up period. Additionally, the proportion of patients with each type of prognosis was categorized by the type of treatment change to compare the proportion of DIP remittance across the types of treatment change.

We assessed the baseline characteristics of the study population, including demographic characteristics (age, sex, type of health insurance, income deciles, and presence of disability) as of the index date, clinical characteristics (Charlson comorbidity index (CCI) within 1 year and Parkinson’s disease within 2 years) before the index date, and the type of offending drugs on the index date [22]. The definition of Parkinson’s disease was established by utilizing ICD-10 code G20 (Parkinson’s disease) for either the primary or secondary diagnosis.

### 2.4. Statistical Analysis

The baseline characteristics of patients with incident DIP and all outcome variables were analyzed descriptively as means, standard deviations, medians, and interquartile ranges (IQRs) for continuous variables, or as proportions for categorical variables. All analyses were performed using SAS Enterprise Guide version 7.1 (SAS Institute Inc., Cary, NC, USA).

## 3. Results

### 3.1. Baseline Characteristics of the Study Population

We identified 1252 patients diagnosed with DIP between 1 January 2004 and 31 December 2013, and 42 patients were excluded because of the presence of a history of DIP diagnosis within two years from the index date. Among them, 272 patients who were prescribed offending drugs to be taken for a period that overlapped the index date were selected as the study population. Approximately half of the selected patients were aged ≥ 60 years, and 62.5% were women (Table 2). The proportion of National Medical Aid beneficiaries among the study population was 7.4%, which was higher than that among the total NHIS-NSC population (2–3%). Parkinson’s disease was diagnosed in 15.4% of the study population. Most patients (95.2%) received GI motility drugs or antipsychotics as the offending drugs.

### 3.2. Treatment Patterns after Incident DIP Diagnosis

**Figure 2:**
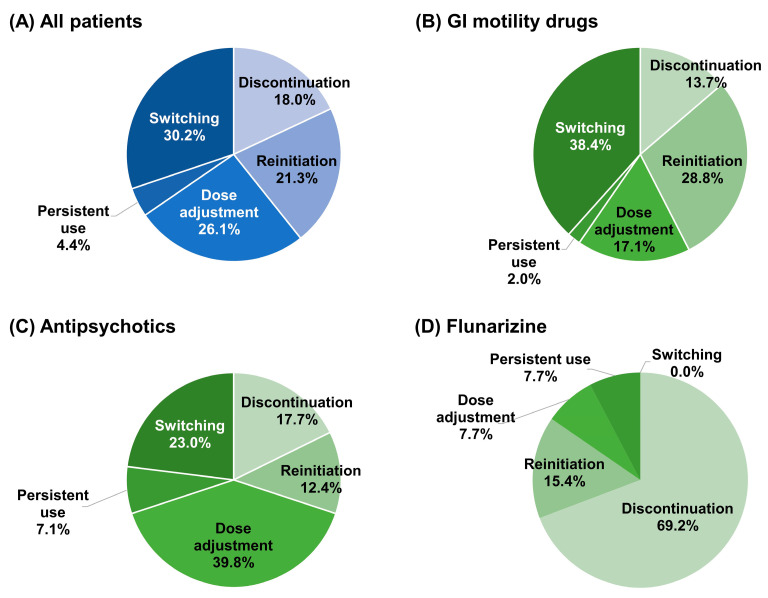
Treatment patterns after the occurrence of incident drug-induced parkinsonism. Abbreviations: GI, gastrointestinal.

Among all the patients, the most common modification after the occurrence of incident drug-induced parkinsonism (DIPs) was switching to other offending drugs (30.2%), followed by dose adjustment (26.1%), reinitiation (21.3%), and discontinuation (18.0%) (Figure 2). Notably, 4.4% of patients consistently received offending drugs. Of the patients who discontinued offending drugs, 69.4% discontinued taking these drugs right after DIP occurrence. More than half of the patients who reinitiated offending drugs chose another drug within the same therapeutic class (56.9%), while 32.8% restarted the same drug at the same dose. Male patients had a higher proportion of discontinuation (20.3% vs. 16.5%) and a lower proportion of reinitiation (19.6% vs. 22.4%) and dose adjustment (24.5% vs. 27.1%) compared to female patients.

Treatment patterns differed according to the type of drug used. Most patients taking GI motility drugs switched to other GI motility drugs (38.4%) or reinitiated drugs after temporary discontinuation (28.8%). Among the patients who reinitiated drugs, 61.9% initiated other kinds of GI motility drugs, while 38.1% initiated the same drug with or without dose adjustment (11.9% and 26.2%, respectively). Patients taking antipsychotics mostly adjusted the dose of antipsychotics (39.8%) or switched to other antipsychotics (23.0%). The proportion of patients who persistently used offending drugs was higher in antipsychotic users (7.1%) than in GI motility drug users (2.0%). Most patients taking flunarizine discontinued the drug (69.2%).

The median time to the first treatment modification was 42.0 days (an IQR of 17.0 to 166.0 days and a mean of 113.3 days), 61.0 days (an IQR of 22.0 to 191.0 days and a mean of 125.2 days), 31.0 days (an IQR of 14.0 to 97.0 days and a mean of 101.9 days), and 25.0 days (an IQR of 10.5 to 153.5 days and a mean of 70.8 days) in all patients, GI motility drugs users, antipsychotics users, and flunarizine users, respectively. Among the patients whose drugs had ever been discontinued during the follow-up period (either temporarily or permanently), the median time to discontinuation was 90.0 days (an IQR of 26.0 to 229.0 days and a mean of 154.8 days), 88.5 days (an IQR of 21.0 to 182.0 days and a mean of 143.4 days), 88.5 days (an IQR of 36.0 to 326.5 days and a mean of 180.6 days), and 149.0 days (an IQR of 56.0 to 158.0 days and a mean of 108.4 days) in all patients, GI motility drug users, antipsychotic users, and flunarizine users, respectively.

### 3.3. Prognosis after Incident DIPs

**Figure 3:**
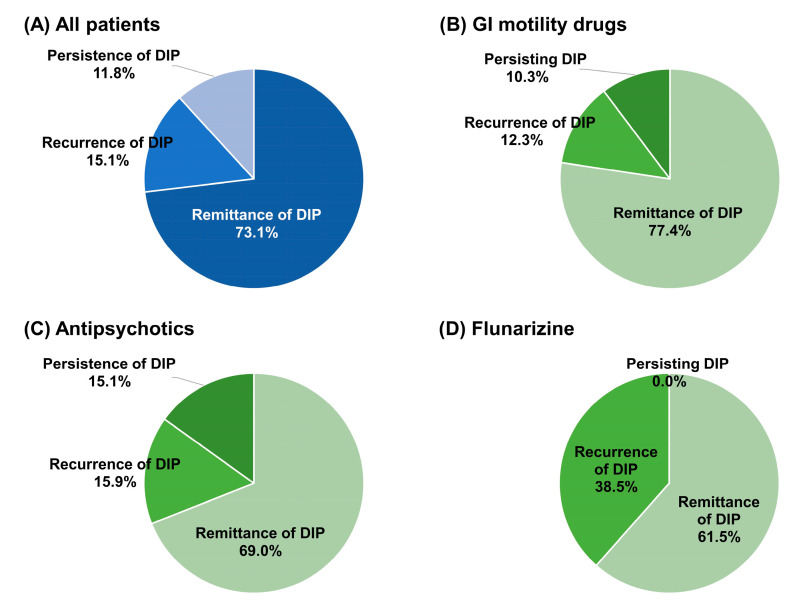
Prognosis after incident drug-induced parkinsonism. Abbreviations: DIP, drug-induced parkinsonism; GI, gastrointestinal.

DIP remittance occurred in more than 70% of all patients, from 61.5% of the flunarizine users to 77.4% of the GI motility drug users (Figure 3). However, approximately 25% of the patients had recurrent or persistent DIP. Male patients had a higher proportion of recurrent (17.6% vs. 13.5%) and persistent (13.7% vs. 10.6%) DIP compared to female patients.

The median time to remittance was 4.0 days (an IQR of 0.0 to 85.0 days and a mean of 70.3 days), 0.0 days (an IQR of 0.0 to 69.0 days and a mean of 62.6 days), 35.5 days (an IQR of 0.0 to 98.0 days and a mean of 86.4 days), and 0.0 days (an IQR of 0.0 to 4.5 days and a mean of 23.1 days) in all patients, GI motility drug users, antipsychotic users, and flunarizine users, respectively. Among the patients who had recurrent DIP, the median time to recurrence was 376.0 days (an IQR of 230.0 to 542.0 days and a mean of 399.8 days), 500.0 days (an IQR of 300.0 to 630.0 days and a mean of 462.1 days), 324.0 days (an IQR of 219.0 to 439.0 days and a mean of 354.5 days), and 210.0 days (an IQR of 209.0 to 427.0 days and a mean of 338.2 days) in all patients, GI motility drug users, antipsychotic users, and flunarizine users, respectively.

**Figure 4:**
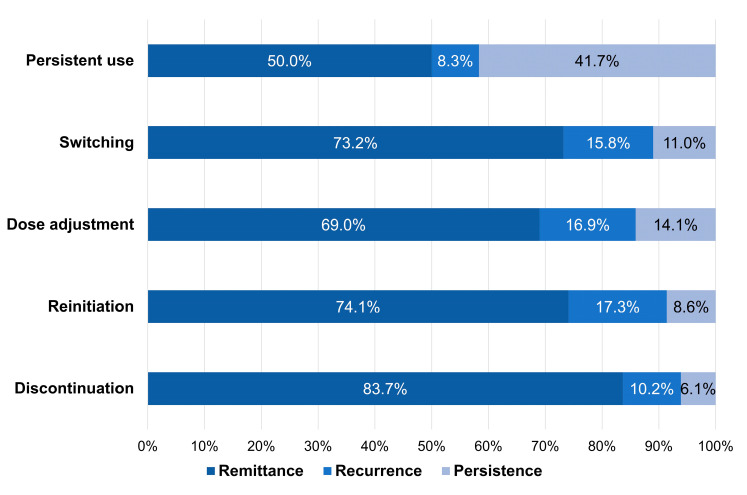
Prognosis by treatment patterns after incident drug-induced parkinsonism.

Figure 4 shows the prognosis for each type of treatment pattern. Among the patients who discontinued the offending drugs, over 80% also showed DIP remittance. However, patients with other patterns had a lower proportion of remittance, especially those who persistently used the offending drugs (50.0%). Among patients who reinitiated offending drugs, patients who chose another drug within the same therapeutic class (75.8%) showed a higher proportion of remittance compared to patients who restarted the same drug at the same dose (63.2%).

## 4. Discussion

This retrospective cohort study explored the treatment changes and prognosis status in patients who had experienced incident DIP in South Korea using a representative national claims database. Among 272 patients with incident DIP, the majority received GI motility drugs or antipsychotics as offending drugs. The patterns of treatment change differed across the types of offending drugs. Patients taking GI motility drugs mostly switched to other GI motility drugs or reinitiated the drugs after temporary discontinuation, while patients taking antipsychotics mostly adjusted the dose of antipsychotics or switched to other antipsychotics. The proportion of persistent users was higher among antipsychotic users than among GI motility drug users. Regarding prognosis, approximately a quarter of patients experienced DIP recurrence or persistence, the occurrence of which was lower in patients who had discontinued the offending drugs than it was in those with other treatment patterns.

This study investigated treatment changes and prognoses among patients with DIP using large, nationwide, and population-based real-world data. Because all citizens in South Korea are covered under either the National Health Insurance program or the Medical Aid program, the NHIS-NSC database could represent the entire Korean population [20]. Herein, we tried to identify patients with definite DIP by defining incident DIP case as an event with a diagnosis code of G21.1 or G25.1, and when the period for which offending drugs were prescribed overlapped with the time of DIP diagnosis. This definition was based on the views of Korean neurologists; therefore, it may reflect the features of the disease in clinical practice [10]. Based on these strengths, we anticipate that our findings may offer valuable insights into predicting DIP in patients prescribed high-risk drugs. Consequently, this could aid in personalized treatment selection for these patients.

In a study using hospital-based data in South Korea, 48% of patients diagnosed with levosulpiride-induced parkinsonism experienced persistent or recurrent parkinsonism even after the withdrawal of levosulpiride [23]. In our study, GI motility drug users showed a lower proportion of DIP recurrence or persistence (22.6%). This estimate is much lower than that of the previous study, considering that the proportion in our study was estimated regardless of the withdrawal of GI motility drugs. The difference might have resulted from the features of the data source because the previous study used data from a large teaching hospital that generally takes care of more severely ill patients compared to those attended to in various hospital settings included in the nationwide data. In addition, levosulpiride entails a high risk of DIP among the GI motility drugs, suggesting its potential impact on the prognosis of DIP [1,6].

Treatment options are lacking for patients experiencing DIP [24]. Although anticholinergic agents or amantadine may alleviate extrapyramidal symptoms, their efficacy has not been established in a large study, and the evidence is conflicting [24,25,26]. Therefore, discontinuing the offending drug may be the best choice if possible [27]. For patients who cannot stop the drug immediately, dose adjustment or switching could be an alternative approach [24]. Appropriate strategies may differ according to the type of offending drug. Given that GI motility drugs are generally used in the short-term and that several alternatives are available for the management of functional dyspepsia, discontinuation or switching to other classes (e.g., histamine type 2 receptor antagonists and proton pump inhibitors) could be considered an effective strategy [28]. Conversely, discontinuing antipsychotics may be unfeasible when considering the risk of exacerbating psychotic symptoms. In this case, dose adjustment or switching to antipsychotics that have a lower propensity to cause DIP would be better approaches than discontinuation [24]. In our previous study, itopride and quetiapine were found to exhibit a comparatively low likelihood of inducing DIP within the respective categories of GI motility drugs and antipsychotics [6]. Nevertheless, the selection of treatment should be based on a thorough assessment of clinical patient features, including the severity of the disease, the presence of comorbidities, concurrent medication usage, and the susceptibility to specific types of adverse events.

Considering the limited evidence on the treatment of DIP, prevention should be the first-line strategy to manage DIP and optimize treatment outcomes in patients prescribed high-risk drugs. Future studies are needed to assess the risk of developing DIP based on patient and drug characteristics. Moreover, our research revealed that approximately a quarter of patients experienced DIP recurrence or persistence. This underscores the importance of healthcare providers monitoring patients with DIP for symptom remission. In order to prevent the recurrence of DIP, healthcare providers should employ effective strategies that take into account the clinical benefits of utilizing drugs, as well as the potential harm of DIP based on individual patient characteristics.

To properly assess the burden of DIP, it is important to understand its association with the risk of developing Parkinson’s disease. A recent study analyzing nationwide healthcare claims data in South Korea found that patients with DIP had a significantly increased risk of developing Parkinson’s disease [16]. This suggests that DIP may be a strong risk factor for the progression of preexisting subclinical parkinsonism to Parkinson’s disease. To fully understand the relationship between DIP and Parkinson’s disease, it would be beneficial to investigate the association between DIP prognosis and the likelihood of developing Parkinson’s disease. This analysis was not feasible in the present study due to the limited number of DIP patients and the short-term follow-up period. Further studies using a larger database encompassing the entire DIP patient population with an extended follow-up period are necessary.

The findings of this study should be interpreted with caution because of the following limitations: The diagnosis codes for defining DIP may not fully capture all patients with DIP. Because we only used the codes explicitly indicating “drug-induced” symptoms, we could not explore the treatment changes and prognoses in some patients with diagnosis codes related to parkinsonism but not specific to DIP or ion those who were undiagnosed. Nevertheless, we attempted to identify patients with definite DIP, considering that parkinsonism not specific to DIP may not be distinguishable from the early signs of idiopathic Parkinson’s disease. We assessed treatment changes and prognoses during the same follow-up period; therefore, the prognosis might have preceded the treatment changes in some cases. However, we assessed the time to treatment changes and prognoses, and the results partly confirm that treatment changes preceded prognoses in most patients. Our findings are limited to GI motility drugs and antipsychotics that are approved in South Korea and may not be generalizable to other drugs in these categories. In particular, certain GI motility drugs such as metoclopramide and domperidone have varying indications and approval statuses across different countries; therefore, the findings regarding GI motility drugs should be interpreted with caution depending on the context. Furthermore, it is noteworthy that the number of DIP patients included in this study is limited, which is due in part to the utilization of the Sample Cohort database, which comprises only approximately 2% of the Korean population. It should be acknowledged that DIP is frequently under-recognized in clinical settings, and the utilization of data collected from the entire population may provide additional insights into the treatment patterns and prognosis of DIP patients. Nevertheless, the Sample Cohort database employed in this study was constructed through a representative sampling process, thereby ensuring the generalizability of our research findings. Lastly, it should be noted that the percentage of patients undergoing treatment changes and prognosis, which was defined based solely on prescription information, may have been subject to either overestimation or underestimation. This is because healthcare claims data lack information on medication adherence, symptoms, and laboratory test results. Despite these limitations, our study offers valuable insights into the current state of DIP management in the real world and highlights areas of unmet need in DIP care. Based on our findings, future research utilizing a larger database with a longer follow-up period is necessary to investigate effective strategies for managing DIP.

## 5. Conclusions

Overall, DIP patients who were taking GI motility drugs primarily switched to alternative GI motility drugs or resumed their medication after temporary discontinuation. Conversely, those who were taking antipsychotics typically adjusted their dosage or switched to alternative antipsychotic medication. Approximately a quarter of patients experienced DIP recurrence or persistence, which were more prevalent in antipsychotic users than in those taking GI motility drugs. To enhance treatment outcomes, it is imperative to assess the risk of developing DIP in patients who are prescribed GI motility drugs or antipsychotics. Additionally, healthcare providers should monitor patients experiencing DIP for symptom remittance and employ effective strategies to prevent its recurrence.

## Figures and Tables

**Table 1:** Definitions of treatment change and prognosis after incident drug-induced parkinsonism.

Category	Definition
**Treatment change in consumption of offending drugs**	
Discontinuation	Absence of new prescription for more than 60 days
Dose adjustment	Altering the dose of the offending drugs
Persistent use	Continued use of the offending drug without any temporary discontinuation, dose adjustment, or switching
Reinitiation	Restarting the discontinued drug, or switching within a therapeutic class after temporary discontinuation
Switching to other offending drugs	Altering drugs within a therapeutic class
**Prognosis of DIP**	
Persisting DIP	Continuation of DIP diagnosis
Recurrence of DIP	Occurrence of DIP diagnosis after a remittance
Remittance of DIP	Absence of new diagnosis of DIP over 4 months

Abbreviations: DIP, drug-induced parkinsonism.

**Table 2:** Baseline characteristics of patients with drug-induced parkinsonism.

Characteristics	DIP Patients (*n* = 272)
Age (years)	
0–19	10 (3.7%)
20–29	28 (10.3%)
30–39	25 (9.2%)
40–49	31 (11.4%)
50–59	37 (13.6%)
60–69	53 (19.5%)
70–79	68 (25%)
80+	20 (7.4%)
Sex	
Male	102 (37.5%)
Female	170 (62.5%)
Type of health insurance	
National Health Insurance program	252 (92.6%)
National Medical Aid program	20 (7.4%)
Income deciles ^1^	
0	21 (7.7%)
1	20 (7.4%)
2	15 (5.5%)
3	18 (6.6%)
4	23 (8.5%)
5	17 (6.3%)
6	23 (8.5%)
7	25 (9.2%)
8	19 (7.0%)
9	36 (13.2%)
10	55 (20.2%)
Disability	
No disability	225 (82.7%)
Mild disability	32 (11.8%)
Severe disability	15 (5.5%)
Comorbidities	
Charlson comorbidity index, mean (SD)	2.2 (2.3)
Parkinson’s disease	42 (15.4%)
Offending drugs	
Antipsychotics	146 (53.7%)
GI motility drugs	113 (41.5%)
Flunarizine	13 (4.8%)

Abbreviations: DIP, drug-induced parkinsonism; GI, gastrointestinal; SD, standard deviation. ^1^ Income deciles were defined based on health insurance contributions. The values 0 and 10 denote the lowest and highest deciles, respectively.

## Data Availability

Not applicable.
